# Serological Prevalence of Contagious Bovine Pleuropneumonia in Niger in 2017

**DOI:** 10.3389/fvets.2018.00238

**Published:** 2018-10-12

**Authors:** Mahamadou Seyni Yansambou, Alpha Amadou Diallo, Moumouni Idi, Haladou Gagara, Abdoul Malick Haido, Rianatou Bada Alambedji

**Affiliations:** ^1^Laboratoire Central de l'Elevage (LABOCEL), Niamey, Niger; ^2^Laboratoire National de l'Elevage et de Recherches Vétérinaires (LNERV), Dakar, Senegal; ^3^Ecole Inter-Etats des Sciences et Médecine Vétérinaires de Dakar, Dakar, Senegal; ^4^Direction des Services Vétérinaires, Niamey, Niger

**Keywords:** contagious bovine pleuropneumonia, serological prevalence, c-ELISA, risk strata, Niger

## Abstract

Contagious bovine pleuropneumonia (CBPP) is a highly contagious disease of cattle caused by *Mycoplasma mycoides* subsp. *mycoides* Biotype Small Colony (MmmSC). The disease currently occurs in most of sub-Saharan Africa and where it is endemic and a major constraint for improving pastoral productivity. Following the persistence of this scourge, and in order to control this disease, a serological survey was conducted to determine the prevalence of CBPP in Niger. In fact, 1,590 sera were collected following a stratified sampling plan based on the risk factor of dissemination of CBPP. The analysis were performed at the Central Livestock Laboratory using the c-Elisa test. The results obtained show a wide distribution of the disease with an overall prevalence of 4.15% at individual level. The highest prevalences were recorded in the South-East regions [Zinder (7.5%), Diffa (7.5%)] and the West part [Tahoua (6.9%)]. The prevalence at the commune level was about 36.55%, which was relatively high. The prevalence at strata level was 36.55% (95% PI 0.2428–0.4882). The expected prevalences did not match those found. The results of this serological survey are considered the reference situation (T0) of CBPP in Niger with the PRAPS project, and allowed to the country to redefine control policies for better control of the disease at national and sub-regional level.

## Introduction

Second activity after agriculture, livestock in Niger represents 11% of national Gross Domestic Product (GDP) and 35% of agricultural GDP. The sale of livestock products places this sector in second position after uranium. This activity provides permanent employment to more than 87% of the population who breed full-time or part-time (1).

The difficulty in practicing this activity is the availability and access to water and pasture. The constraints are also marked by an increased insufficiency of sanitary surveillance linked to the lack of a reliable supply chain for pharmaceutical and zoo-technical products. Although some epizootic diseases such as rinderpest have been eradicated, some sanitary constraints are now a concern as they could significantly compromise the development of animal resources in Niger. The most common diseases are parasitic diseases and epizootic diseases such as the Contagious Bovine pleuropneumonia (CBPP). This disease afflicts heavy loss in the cattle herd in Niger.

CBPP is an infectious, contagious, inoculable disease caused by mycoplasma, *Mycoplasma mycoides* subsp. *mycoides* Biotype Small Colony (MmmSC). It is one of the most threatening diseases of cattle herds in sub-Saharan Africa since rinderpest, which was the most deadly disease of the species a few years ago, has been eradicated (2).

CBPP has been enzootic for several years in Niger and causes losses that are difficult to evaluate because of its insidious evolution. Formerly under control thanks to the vaccination campaigns with mixed vaccine against rinderpest and CBPP, the pleuropneumonia has experienced an upsurge since the cessation of rinderpest vaccination. Unfortunately, control efforts have been slackened with outbreaks of cases.

The fight against this disease in sub-Saharan Africa is made difficult by the low efficiency of available vaccines and by the impoverishment of states that cannot implement the systematic slaughter of contaminated herds or that cannot control the movement of livestock (3).

The detection of infected animals within the cattle herd is a prerequisite for any successful fight against CBPP. According to Provost et al. (2), the eradication of CBPP follows three main phases: serological screening of infected animals, the slaughter of serologically positive animals and the maintenance of serological epidemiological surveillance (2).

The main problems for control or eradication are the frequency of subacute or subclinical infections, the persistence of chronic carriers after the clinical phase and the lack of extended vaccination coverage. Control strategies are based on early detection of outbreaks, control of animal movements and a slaughter policy. The implementation of these strategies has led to the eradication of the disease in North America and Europe. In Africa, disease control is currently focused mainly on immunization campaigns, but the prevalence of the disease in several African country like Niger is not very well known.

The serological diagnostic is one of the first ways to get information on the prevalence and have a good surveillance of the CBPP.

To assess the effectiveness of vaccination operations in Niger, it is necessary to follow the evolution of the disease (its decay) over time through a serological prevalence proposed by this T0 serological survey.

## Materials and methods

### Study area

In Niger, livestock farming is practiced by nearly 87% of the active population either as a main activity or as a secondary activity after agriculture.

Livestock is the dominant form of savings for rural and urban households, helping to build resilience in response to particular crises and social events.

Niger is a vast Sahelian country of 1,267,000 km^2^, located in West Africa; limited to the North by Algeria and Libya, to the South by Nigeria and Benin, to the East by Chad and to the West by Mali and Burkina Faso. The territory is divided into eight (8) regions, sixty-seven (67) departments and two hundred and sixty-five (265) communes divided into fifty-two (52) urban communes and two hundred, and thirteen (213) rural communes.

### Sampling procedure

The sampling protocol focused on a stratified design of the risk (risk of occurrence of the disease) that reduces the sample size to constant precision and thus the cost of the surveys compared to a random sample design simple. Four strata were defined according to the probability of occurrence resulting from the crossing of the risk of emission and risk of diffusion of the disease. This is n1 = negligible risk; n2 = low risk, n3 = high risk and n4 = very high risk. The epidemiological unit chosen was the commune.

As strata were defined, the epidemiological units are then randomly drawn in strata by simple random sampling, with a number proportional to the weight of each stratum.

Based on the risk of CBPP in the dry season, 265 communes are classified as follows:

- Negligible risk stratum, with an expected prevalence rate P1 of 10% of the infected communes. There are 19 communes in this stratum (7%);
- Low risk stratum, with an expected prevalence rate P2 of 25% of the infected communes. There are 56 communes in this stratum (21%);
- High risk stratum, with an expected prevalence rate P3 of 50% of the infected communes. There are 140 communes in this stratum (53%);
- Very high risk stratum, with an expected prevalence rate P4 of 80% of the infected communes. 50 communes have been identified in this stratum (19%).

**Figure 1:**
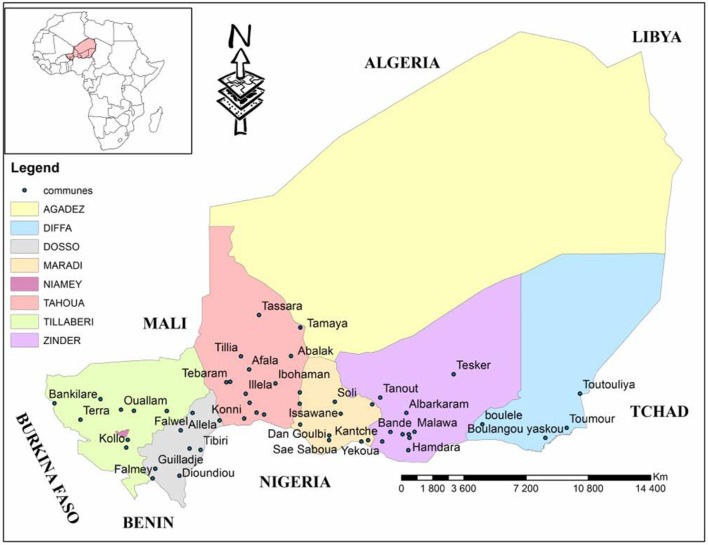
Geographical representation of the regions of Niger with selected communes of the study (4).

In each of the selected communes, a meeting on animal health with breeders was organized. During this meeting, they received information on CBPP and we asked if their animals have recently experienced an episode of CBPP. We selected these breeders and we organized a sampling session in their herds. The selected communes were represented in Figure 1.

At the level of each selected Epidemiological Unit (EU), animals were specifically targeted within the herds by first selecting those who have had respiratory problems in the last two years. The older animals were completed to get 30 samples in the EU if the first criteria animals were not reach the expected number per EU (30 animals).

If the breeders have not known any clinical suspicion of CBPP, then the oldest females are chosen because they have had the best chance of being confronted with mycoplasma.

**Figure 2:**
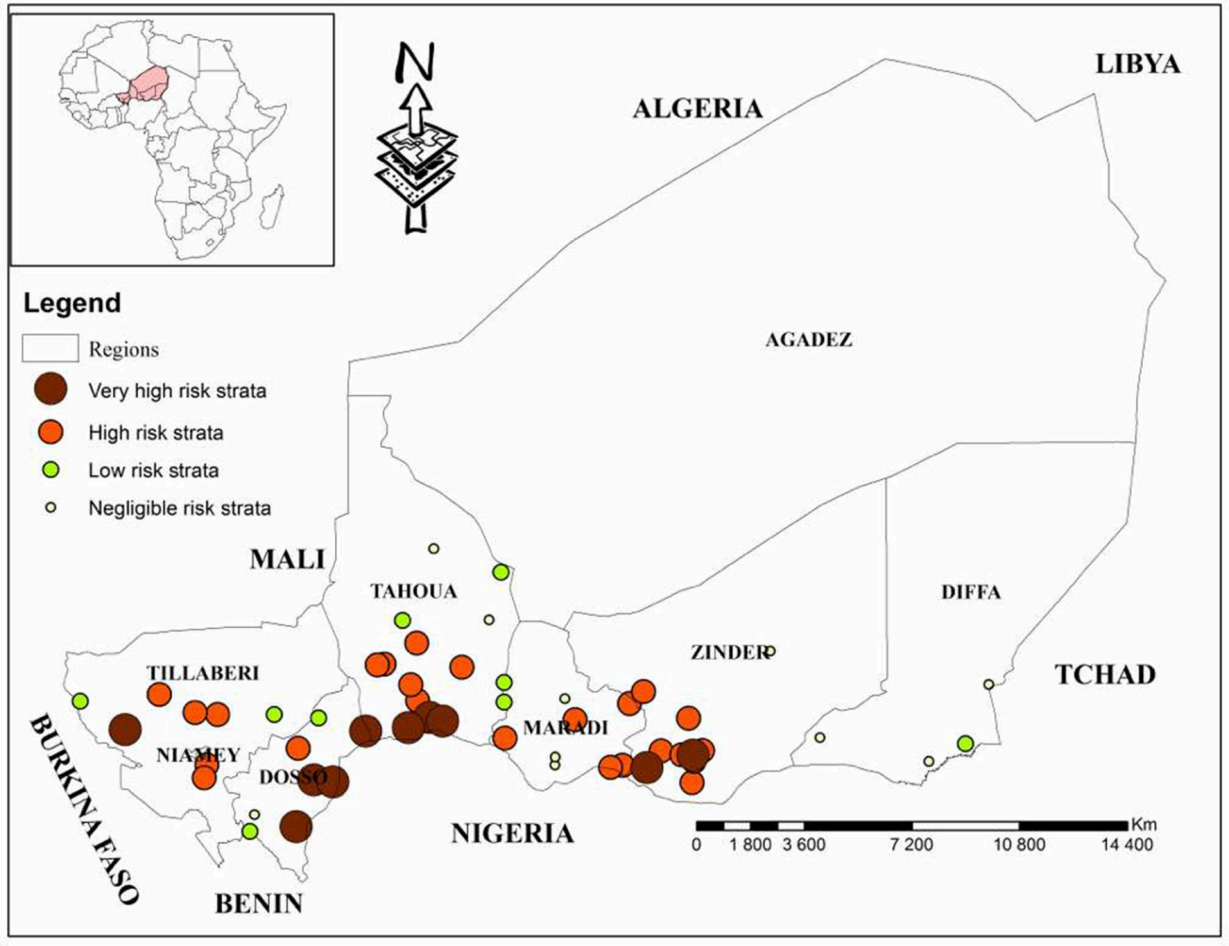
Geographical representation of strata according to the risks of occurrence of CBPP (4).

Fifty three (53) EU were identified in Niger (Table 1) by this analysis, divided into 4 areas, 10 of which are very high risk, 24 of high risk, 9 of low risk and 10 of negligible risk (Figure 2). Thus, 1590 sera were collected for this study.

**Table 1:** Presentation of EU (communes) numbers by region and stratum.

**Regions**	**Negligible risk Stratum**	**Low risk Stratum**	**High risk Stratum**	**Very High risk Stratum**	**Total**
Agadez	0	0	0	0	0
Diffa	3	1	0	0	4
Dosso	1	2	1	3	7
Maradi	3	2	3	0	8
Niamey	0	0	0	0	0
Tahoua	2	2	6	4	14
Tillabéri	0	2	5	1	8
Zinder	1	0	9	2	12
Total	10	9	24	10	53

In the respect of ethics, our study didn't require the ethics committee approval in accordance with local legislation.

The analysis of the samples was carried out from February 27 to March 10, 2017 at Laboratoire Central de l'Elevage (LABOCEL) Niamey (Niger).

### Serological tests

The serological analysis was performed by IDEXX c-ELISA version P05410/10 kit for contagious bovine pleuropneumonia, which was acquired from CIRAD/Institut Pourquier (Montpellier, France), with a specificity around 99.7% and high sensibility.

Following the manufacturer's instructions, each serum was diluted and mixed with specific anti-MmmSC antibody named Mab 117/5.

The mixture was incubated in an MmmSC lysate-coated plate followed by incubation with peroxidase substrate solution after incubation with the peroxidase-labeled anti-mouse IgG antibody.

Reading was performed in a spectrophotometer “ThermoScientific Multiskan FC” at 450 nm. The percentage of inhibition (PI) for each serum was calculated as follows:

$$\begin{array}{l} \text{PI }=\;100\text{x}\left[\left(\text{ODCm}-\text{ODTest}\right)/\left(\text{ODCm}-\text{ODCc}\right)\right] \end{array}$$

Where OD = optical density, Cm = monoclonal control, Test = test serum, and Cc = conjugate control. The validity criteria were an OD between 0.5 and 2.0 for Cm; an OD below 0.3 for Cc.

In total, 19 plates were used to make analysis. For the calculation of the measurement uncertainty which is equal to (standard deviation of individual C + values) x2, we used instead the results of the “Internal Reference Material” (MRI), the results of the control weakly positive (C +) of the 19 plates.

After the calculations we obtained: 3.9 × 2 = 7.8 as measurement uncertainty. We considered the measurement uncertainty at the threshold established in our laboratory for c-ELISA PPCB δi = ±8. The IDEXX commercial kit has a cut off of ɼ = 50%, and our cut-off was ɼ + δi = 50 + 8 = 58%.

A PI of negative control was lower than 50%; a PI positive close to the threshold control between 50 and 58%; and a PI of positive control equal to or greater than 58%. Thus, a test serum with a PI equal to or greater than 50% was considered positive.

### Data analysis

The CBPP individual prevalence (prevalence of anti-MmmSC antibodies) was estimated by comparing the number of positive sera to the number of sera tested.

The strata prevalence was estimated by relating the number of positive municipalities to the number of communes in the stratum considered.

A commune is positive if at least one animal is positive or two animals are doubtful. Otherwise, it is negative.

The calculation of the prevalence, variance and standard deviation for a stratified survey design with H strata, of size *N*_1_,…., *N*_*h*_,…*N*_*H*_ (total population size: *N*), the estimate of the prevalence rate is (5, 6):

$$\begin{array}{l} \hat{p_{H}}=\overset{H}{\underset{h=1}{\sum }}\frac{N_{h}}{N}p_{h} \end{array}$$

*P*_*h*_ = *m*_*h*_ / *n*_*h*_ is the prevalence rate in stratum *h*

*m*_*h*_ is the prevalence in stratum *h*, that is, the number of positive units in stratum *h*

*n*_*h*_ is the sample size for stratum *h*.

## Results

The serological survey was conducted on 53 EU. The number of serological tests performed was 1590.

**Figure 3:**
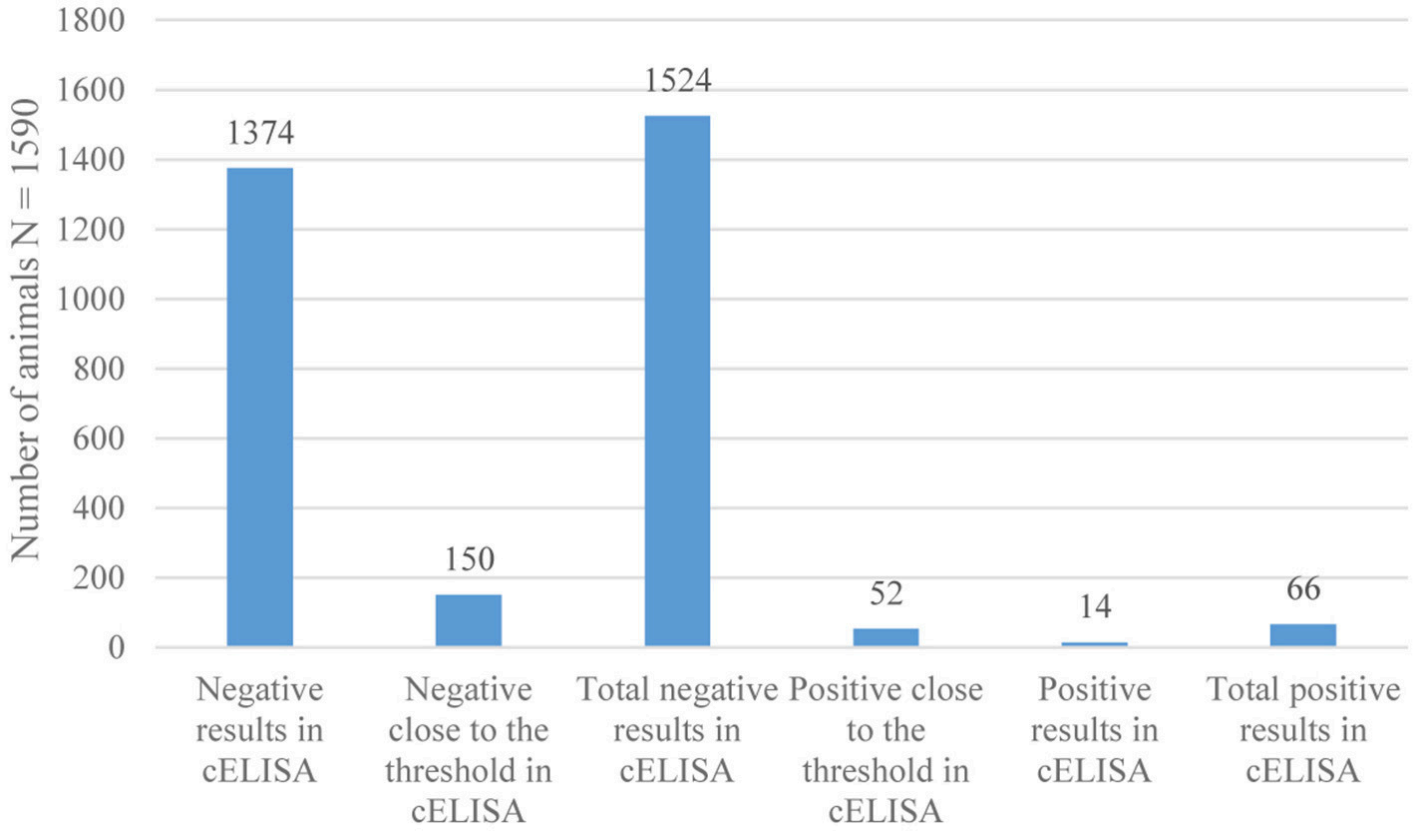
Results of serological tests of CBPP by c-ELISA.

The measurement uncertainty at the threshold level established in our laboratory was ± 8. A serum with a PI (percentage of inhibition) between 42 and 50 was considered “negative close to the threshold” and a serum with a titer between 50 and 58 as “positive near the threshold.” The sera with PI < 42 were “negative”, and PI > 58 were “positive” (Figure 3).

From the 1590 sera, 66 were tested positive, giving an individual serological prevalence of CBPP of 4.15% at 95% with a confidence interval of [3.17–5.13%].

The results show that the Diffa, Zinder and Tahoua regions have the highest prevalences respectively 7.5; 7.5, and 6.9%.

The region of Dosso was less affected with a prevalence of 0.47%.

In Tillabéri and Maradi, the prevalence is zero because from the 240 sera tested in these regions, none has been positive (Table 2).

**Table 2:** Individual Serological prevalence of CBPP in Niger by Region.

**Regions**	**Number of Samples**	**Number of seropositive**	**Individual prevalence % 95% PI**	***P*-value**
Diffa	120	9	7.50% [2.76%−12.23%]	<0.0001
Dosso	210	1	0.47% [0%−1.41%]	
Maradi	240	0	0%	
Tillabéri	240	0	0%	
Tahoua	420	29	6.9 % [4.47%−9.33%]	
Zinder	360	27	7.5% [4.77%−10.22%]	

The difference of CBPP individual seroprevalence between regions was statistically significant (*p* < 0.05).

The situation of CBPP, according to the surveyed communes was summarized in Table 3 by stratum. Analysis of results by stratum revealed the following information.

**Table 3:** Communes status results per strata.

**Strata**	0^*^	1^*^	9^*^	**Total**	**Commune status**
High Risk	693	5	22	720	
Afala	27	1	2	30	1
Albarkaram	27	1	2	30	1
Bande	29		1	30	0
Barzanga	27		3	30	1
Dan Goulbi	30			30	0
Dingazi banda	30			30	0
Dungass	29		1	30	0
Edir	29		1	30	0
Falwel	30			30	0
Gafati	28		2	30	1
Gangara	28	1	1	30	1
Hamdallaye	30			30	0
Hamdara	28	1	1	30	1
Issawane	30			30	0
Kantche	30			30	0
Kollo	30			30	0
Koona	30			30	0
Kossori	28	1	1	30	1
Malawa	27		3	30	1
Ouallam	30			30	0
Tanout	29		1	30	0
Tarjamatt	27		3	30	1
Tondikiwindi	30			30	0
Toumboul	30			30	0
Low Risk	261	3	6	270	
Alamboule	30			30	0
Azagor	30			30	0
Birni Lallé	30			30	0
Falmey	30			30	0
Gawai	28	1	1	30	1
Kourfey centre	30			30	0
Soucoucoutane	29		1	30	0
Tamaya forage	28		2	30	1
Toumour	26	2	2	30	1
Negligible risk	280	5	15	300	
Abalak	25		5	30	1
Guilladje	30			30	0
Tarissadat	30			30	0
Tesker	20	3	7	30	1
Boulangou	29	1		30	1
Boulélé	30			30	0
Saé Saboua	30			30	0
Sharkin Haoussa	30			30	0
Soli	30			30	0
Toutouliya	26	1	3	30	1
Very High Risk	290	1	9	300	
Bouta	26	1	3	30	1
Dioundiou	30			30	0
Dogo dogo	29		1	30	0
Guidan dilli	28		2	30	1
Guidan saidi	28		2	30	1
koré Mairouwa	30			30	0
Massalata	30			30	0
Tera	30			30	0
Tibiri	30			30	0
Yekoua	29		1	30	0
Total general	1524	14	52	1590	

NB, An animal is categorized:

* *CBPP positive (1) if the PI≥58*.

* *CBPP negative (0) if PI < 50*.

* *CBPP doubtful (9) if 50 ≤ PI < 58*.

A commune is positive if at least one animal is positive or two animals are doubtful. Otherwise, it is negative.

The results at stratum level are as follows (Table 4):

- For the negligible risk stratum, with an expected prevalence rate of 10% of the infected communes, we obtained a prevalence of 30% [0–60.65%].
- Low-risk stratum showed a prevalence of 33.33% [0–66.77%], while the expected prevalence was 25% of the infected communes.
- A prevalence of 37.5% [17.24–57.75%] was obtained for the high-risk stratum, whose expected prevalence rate of the infected communes was 50%.
- For the very high risk stratum, with an expected prevalence rate of 80%, we had a prevalence of 40% [7.23–72.76%].

**Table 4:** Strata Serological prevalence of CBPP in Niger.

**Strata**	**Number of communes**	**Sum of communes status**	**Number of commune per strata**	**Prevalence by strata at 95% CI**	***p*-value**
High risk	24	9	140	37.5% [17.24%−57.75%]	0.964
Low risk	9	3	56	33.3% [0%−66.77% ]	
Very high risk	10	4	50	40% [7.23–72.76%]	
Negligible risk	10	3	19	30% [0%−60.65%]	
Total general	53	19	265	35.8% [22.5%−49.19%]	

The difference of CBPP prevalence between strata was not statistically significant (*p* > 0.05).

The analysis of data through CIRAD excel worksheet allowed to report a total prevalence based on the risk approach of 36.55% [24.84–48.82%] (Table 5).

**Table 5:** Epidemiological parameters.

**Parameter**	**Value**	**Value %**
Strata prevalence	0,36553459	36.55%
Strata variance	0,00391844	0.39%
Strata standard deviation	0,06259744	6.26%
Lower bound 95% CI	0,24284361	24.84%
Upper bound 95% CI	0,48822557	48.82%

## Discussion

To get unvaccinated animals, our study was conducted at the beginning the dry season, referring to the vaccination campaign in Niger which starts at late dry season and early rainy season.

The choice of the communes in each EU on the spot was not easy, given the poor apprehension of the CBPP by the breeders.

The OIE reference technique for CBPP serology was the complement fixation test (CFT). This technique has been of great service in the past for the eradication of CBPP in many countries.

It has some disadvantages, notably the difficulty of standardizing the production of the antigen or the presence of a certain number of non-specific positive reactions (7).

The CFT was considered the most sensitive and most specific standard tests despite its limitations(8). To improve the diagnostic accuracy of CBPP, a competition ELISA has been developed and would appear to be better adapted to diagnosis by a better choice of monoclonal antibody (Mab) very specific to MmmSC (9, 10).

Whether for the CFT or the c-ELISA test, it must be remembered that the results obtained can only be used for a sanitary interpretation of a herd. Indeed, animals in incubation cannot be detected, as many animals in the chronic phase of the disease because the percentage of positive animals decreases with time.

The use of both tests in common (c-ELISA and CFT) is more sensitive than each individual serological test. But for the sake of economy, when only one serologic test alone should be used for routine screening, c-ELISA will be recommended rather than CFT (11).

Vaccination with T1/44 or T1-sr strains does not systematically lead to the appearance of antibodies in vaccinated animals. The CFT or the c-ELISA test cannot be used to monitor the effectiveness of vaccination campaigns. However, since post-vaccine antibodies do not persist for more than 3 months, this c-ELISA test can be used for the detection of outbreaks, even in areas where vaccination is practiced (10).

In order to be sure that the MmmSC antibodies detected during the study resulted from natural infection, sampling was based on unvaccinated animals. Therefore, the positive results obtained in this study reflect the presence of specific antibodies after natural infection.

The study of the prevalence of CBPP in Niger is an important starting point in the development of an adapted control strategy.

Thus, the results of the study show that the overall individual prevalence of 4.15% [3.17–5.13%] is relatively low. But there is a fairly wide distribution in the country.

From the 53surveyed communes, 19were infected, giving a prevalence of 35.8% [22.5–49.19%] and 4 were positive out of 6 surveyed regions, a prevalence of 66.66% [12.47–120.86%].

These results were slightly higher than those found by Bloch and Diallo in 1991. During this work, 400 sera were collected, all from unvaccinated animals throughout the country. Fifteen were positive, corresponding to a serological prevalence of 3.7%. From these, 10 were raised in transhumant system(nine in the department of Tahoua and one in Diffa) (12).

This difference could be explained by the negligible number of samples, but also the random selection of a sub-sample (400 sera) of unvaccinated animals by the fact that the collection of sera took place 1 month after the vaccination campaign against rinderpest and pleuropneumonia. The difference of prevalence could also be explained by the fact that this study used CFT unlike our study which used c-ELISA as a serological test.

In 2012, Senegal lost its status as a country provisionally free from contagious bovine pleuropneumonia, following an outbreak during this year in the region of Tambacounda. Subsequently, other outbreaks were confirmed in the regions of Kolda and Matam respectively in November 2013 and at the end of December 2013 (13).

A recent study has shown that in Senegal, out of 2561 sera sampled, 115 tested positive for CBPP corresponding to a serological prevalence of 4.49% [3, 68–5.29%] at individual level (14). This prevalence is similar to our results.

Our results were lower to those of Séry et al. in Mali where 8007 sera samples were routinely collected from 199 herds of cattle throughout the country. The results showed a national prevalence of 18.11% at the individual level and 85.93% at the herd level (15). This difference can be explained by the sampling method. In each circle, four villages whose one herd per village, were randomly selected and 40 sera by herd) which is not the same as that used during our study. The circle was all most similar to our commune, we had only one herd with 30 sera samples per commune whereas they had four herd with 40 sera samples.

The regions of high prevalence (Diffa and Zinder) are all border with Nigeria in the southern part, and Tahoua is also border with Mali in its northern part. These three regions have a very high density of cattle, about 50% of the national livestock.

These two borders are all known by their insecurity, which leads to the inaccessibility of veterinary agents and vaccinators in these areas.

In agro-pastoral areas of Nigeria, the prevalence of CBPP at the flock level was 54.7%, and the proportion of animals with a monoclonal antibody MmmSC detected using of c-ELISA, was 30.2% (16).

The regions of Dosso, Tillabéri and Maradi have the lowest prevalence respectively 0.47, 0, 0%.

The regional variations of the CBPP prevalence were probably linked to the farming methods and climatic, hydrographic and insecurity conditions prevailing in different regions.

In the Tillaberi and Dosso regions, one might expect the presence of the river suitable for agriculture and livestock, in addition to the vast areas of pastures and many water points which are permanently frequented by livestock, to raise the highest positive serology rate in these areas. But this is not the case. This rate is 0% in Tillaberi and 0.47% in Dosso.

Indeed, for economic, social and cultural reasons, the most widespread breeding method in this zone of Niger is extensive and transhumant, we can then think that most of the animals were moving toward Burkina Faso and Benin, two border countries where fodder was in large quantity at the time of sampling.

It is also important to emphasize the importance of chronic carriers in which no detectable antibodies exist even with sensitive serological tests.

From the 4 strata sampled (the high risk strata, the low risk strata, the very high risk stratum and the negligible risk stratum) we had prevalence rates of 37.5% [17.24–57.75%], 33.3% [0–66.77%], 40% [7.23–72.76%] and 30% [0–60.65%]. (Table 6)

**Table 6:** Comparison of expected and real prevalence at strata level.

**Label**	**Expected prevalence**	**Real prevalence**
High risk	50%	37.5%
Low risk	25%	33.3%
Very high risk	80%	40%
Negligible risk	10%	30%

Among these results, the prevalence rates for the low-risk and negligible-risk strata were higher than the expected values. In contrast, the prevalence rates of high-risk and very high-risk strata were lower than expected.

This situation can be explained by the fact that the disease distribution is more or less homogeneous on the southern part of the whole country from east to west.

Furthermore, the CBPP strata prevalence difference between regions was not statistically significant with a *p*-value equal to 0.964 > 0.05.Which further supports a statistically homogeneous distribution.

Niger has for many years been carrying out an annual and routine vaccination campaign for all animals over 6 months of age, using a bivalent vaccine against rinderpest and contagious pleuropneumonia. Following the eradication of rinderpest in Niger, the bivalent vaccine was abandoned in favor of another CBPP vaccine, the T1sr strain.

Despite the yearly vaccination, there was an increasing number of CBPP cases in Niger. This can be explained by the fact that some areas were inaccessible for vaccination because of insecurity, but also the unwillingness and refusal of some farmers against this vaccination although it is completely free.

Agro-climatic characteristics determine the spatial distribution of pastures and water points, which in turn determine the movement and aggregation of herds.

In particular, there is evidence in the field that animal movements are closely related to forage density on available pastures. This is why the prevalence is not geographically homogeneous.

According to the models presented by Mariner et al. (17), the probability of infection of a herd should be positively related to the contact rate between this herd and other herds (17). Livestock practices are the main determinants of contact rates between herds. In particular, it seems clear that transhumant herds have more contact with other herds than sedentary herds. This is the reason why transhumant livestock is suspected to be largely responsible for the maintenance and spread of the disease within and between neighboring countries in Africa (2, 18, 19).

Finally, herds are open entities that frequently exchange animals (17). According to Provost (2) and Masiga et al. (18), nomadism and animal trade would be responsible for the maintenance and spread of the disease in one country and between neighboring countries(2, 18).

On the basis of the results obtained, we can say that CBPP is an endemic disease in Niger. The movement of cattle in recent years may be the cause of the re-emergence of this disease in the sub region. The current situation could thus constitute a serious obstacle to the development of livestock farming in sub-Saharan countries.

Knowledge of the prevalence and distribution of CBPP at the national level should lead us to make recommendations for limiting its progression and also for defining strategic axes for the eradication of the disease at national and sub-regional level.

## Conclusion

Contagious Bovine Pleuropneumonia is an endemic disease in Niger. To make a situation of the disease, a seroprevalence study have been conducted in the country. The results of this study gave an individual prevalence of 4.15 and 36.55% at strata level. Which allow to make a true situation of the disease. These data give an overview on the serological prevalence of the CBPP and to further the knowledge on the disease. Thus, this step can be considered as a reference situation T0 at the national level on the basis of the risk analysis. Base on that, the implementation of an effective vaccination policy with a sustainable sero-monitoring system and if it possible, animals movement control; will allow a global control of CBPP.

## Ethics statement

The handling of the animal was made in strict respect of the animal welfare and all the samples carried out with consents of the cattle breeders. For this, awareness days were organized by the officers of the ministry of Livestock.

## Author contributions

MY: Principal author, the article as part of my PhD in Biotechnology and Animal Health (Bacteriology). AAD: Supervision for laboratory analysis and article writing. MI: Collection of sera used for the analysis of seroprevalence. HG: Head of the serology department who analyzed and validated the serological results. AH: General Director of Animal Health, administrative support for the compilation of data. RA: Ph.D. surpervisor, she validated the work before submission.

### Conflict of interest statement

The authors declare that the research was conducted in the absence of any commercial or financial relationships that could be construed as a potential conflict of interest.
